# A Simple Method for Removal of Carbon Nanotubes from Wastewater Using Hypochlorite

**DOI:** 10.1038/s41598-018-38307-7

**Published:** 2019-02-04

**Authors:** Minfang Zhang, Yinmei Deng, Mei Yang, Hideaki Nakajima, Masako Yudasaka, Sumio Iijima, Toshiya Okazaki

**Affiliations:** 1 National Institute of Advanced Industrial Science and Technology (AIST), 1-1-1 Higashi, Tsukuba, Ibaraki, 305-8565 Japan; 2 Faculty of Science & Technology, Meijo University, 1-501 Shiogamaguchi, Tempaku-ku, Nagoya, 468-8502 Japan

## Abstract

Carbon nanotubes (CNTs) have been applied in a wide range of fields, such as materials, electronics, energy storages, and biomedicine. With the rapid increase in CNTs industrialization, more and more CNT-containing wastewater is being produced. Since concerns about the toxic effects of CNTs on human health persist, CNT-containing wastewater should not be released into the environment without purification, but no effective methods have been reported. In the present study, we report a simple method to eliminate CNTs from industrial or laboratorial wastewater using sodium hypochlorite. Direct treatment of aqueous dispersions with sodium hypochlorite solution completely degraded CNTs into carbon oxides or carbonates ions. Since hypochlorite is environmentally friendly and frequently used as a disinfectant or bleaching agent in domestic cleaning, this method is practical for purification of CNT-contaminated industrial wastewater.

## Introduction

Due to their unique porous structures and outstanding properties, including tremendous flexibility, light weight, high strength and extremely high electrical and thermal conductivity, carbon nanomaterials (CNM) such as carbon nanotubes (CNTs)^1,2^, nanohorns^3^ and graphene^4^ have been explored for various applications in the fields of electronics, materials, and even nanomedicines^5–8^. The number of CNM-containing products appearing in the market is therefore increasing rapidly. By 2015, there were over 100 CNT-related companies worldwide, and the number is increasing year by year. Annual global production of CNTs is estimated to be in the order of hundreds of tons^9^. However, accompanying the expansion of the CNT industry is an increasing amount of CNT-containing industrial wastewater that represents a significant threat to the natural environment if discarded. The release of CNT-containing waste water has the potential to damage aquatic life in water columns and sediment compartments^10^ as well as human health^11–13^. CNT exposure affects cell viability and algal growth^14^ and CNTs can act as pesticide carriers affecting fish survival, metabolism, and behavior^15^. Moreover, toxicology studies have shown that some CNTs might have harmful effects on human health^11–13^. Compared with micrometer-sized carbon-based particles such as carbon black, multi-walled carbon nanotubes (MWNTs) of similar size induce pulmonary inflammation at a lower dose in animal studies, and inhalation exposure of MWNTs induces malignant mesothelioma in mice, suggesting that MWNTs may pose a similar hazard to asbestos^12^. Damage to other organs due to pulmonary exposure to MWNTs has also been reported in animal studies^12,13^. Although most other CNTs are reported to have low acute toxicity, long-term safety is yet to be clarified^16–20^. To protect the environment and mitigate health problems caused by CNTs in water, they must be removed from industrial wastewater before it is released into the environment. It is easy to remove most CNTs from aqueous solution by filtration, but a few types may pass through the filter pores and enter the environment.

To date, no effective method has been ported for the removal of CNTs from waste systems or other liquids. In general, CNTs are chemically extremely stable substances that are difficult to degrade other than by strong acid or oxidation treatment at high temperature, and such methods are not suitable for their removal from aqueous systems. Recent studies show that CNTs can be degraded by enzymatic oxidation using horseradish peroxidase (HRP) and myeloperoxidase (MPO) from human neutrophils^21–24^. The biodegradation mechanism of CNTs is thought to involve oxidation by hypochlorous acid that is generated during enzymatic reaction^19–22^, and sodium hypochlorite or hypochlorous acid have since been shown to oxidize and degrade CNTs^24–28^. Interestingly, 2D graphene oxide sheets are degraded faster than 1D oxidized CNTs^29^, and the wall thickness of CNTs might be not a crucial factor for their degradation by hypochlorite^30^. However, it is still not obvious whether CNTs are completely degraded. In the present study, we demonstrate that hypochlorite can completely remove CNTs from aqueous solutions, suggesting a potential method for the removal of CNTs from CNT-containing industrial wastewater. Sodium hypochlorite and hypochlorous acid are inexpensive and environmentally friendly oxidizing agents that are commonly used as household bleaching or sterilizing agents, suggesting they may be suitable for application in CNT industries and research laboratories.

## Results

**Figure 1:**
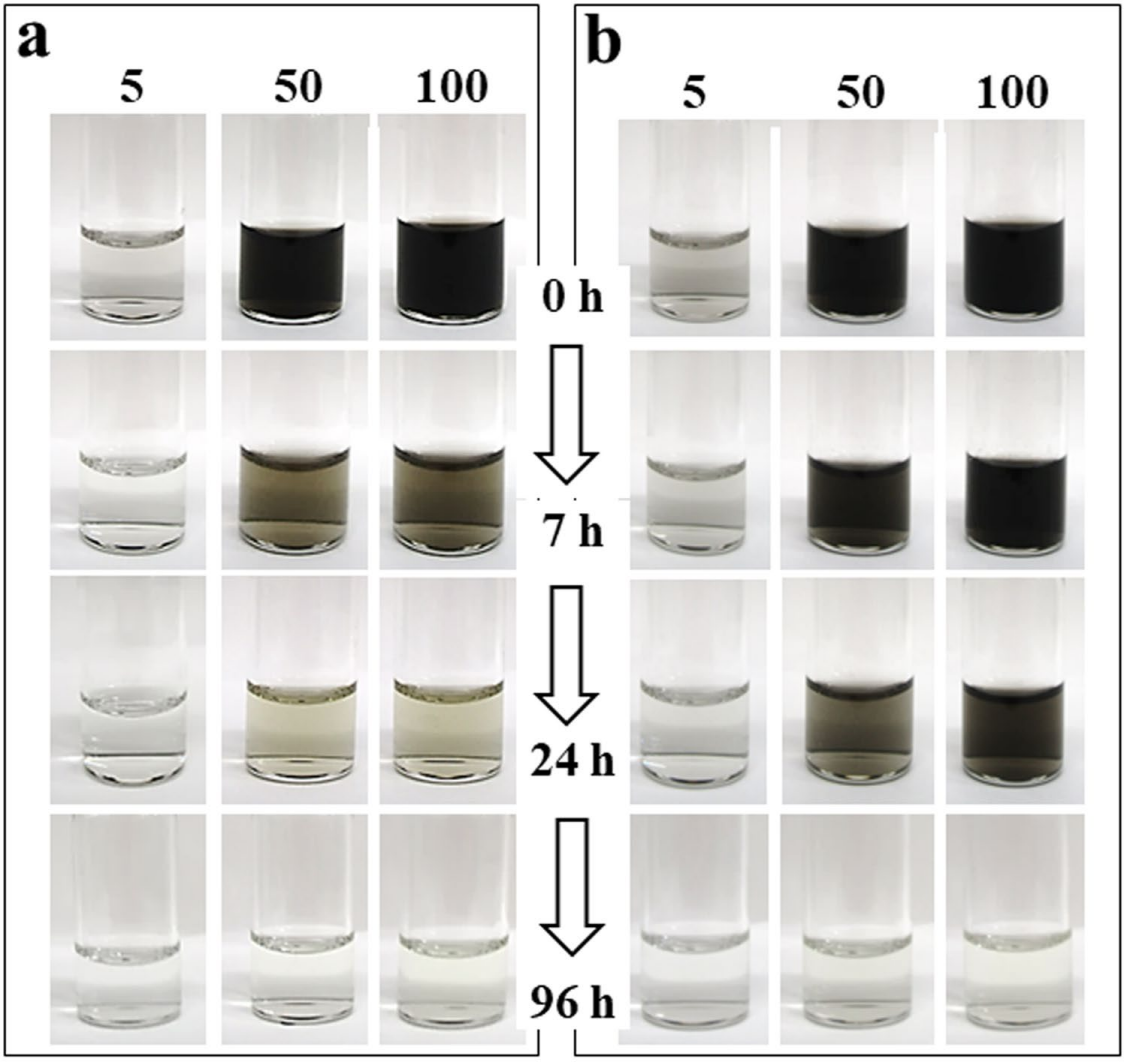
Images of dispersions of single-walled nanotubes (SWNTs; **a**) and multi-walled nanotubes (MWNTs; **b**) after treatment with sodium hypochlorite solution for 0–96 h.

As a demonstration, we show that two typical commercially available CNTs, single-walled carbon nanotubes (SWNTs; SG CNT) and MWNTs (NC 7000), could be completely degraded by treatment with NaClO. To obtain aqueous dispersions, SWNTs or MWNT were pretreated with acids (see Experimental Methods below) and washed with water. The concentrations of CNTs dispersed in water were 5, 50 and 100 mg/L. To monitor the elimination of CNTs from solution, CNT dispersions were mixed with NaClO (1.1%, w/w) aqueous solution and incubated at 37 °C for 1–120 h. The black color of dispersions of SWNTs (Fig. 1a) or MWNTs (Fig. 1b) faded gradually with increasing treatment time, and 5 mg/L dispersions of SWNTs became completely transparent after 24 h, while 50 mg/L and 100 mg/L dispersions became completely transparent after 96 h.

**Figure 2:**
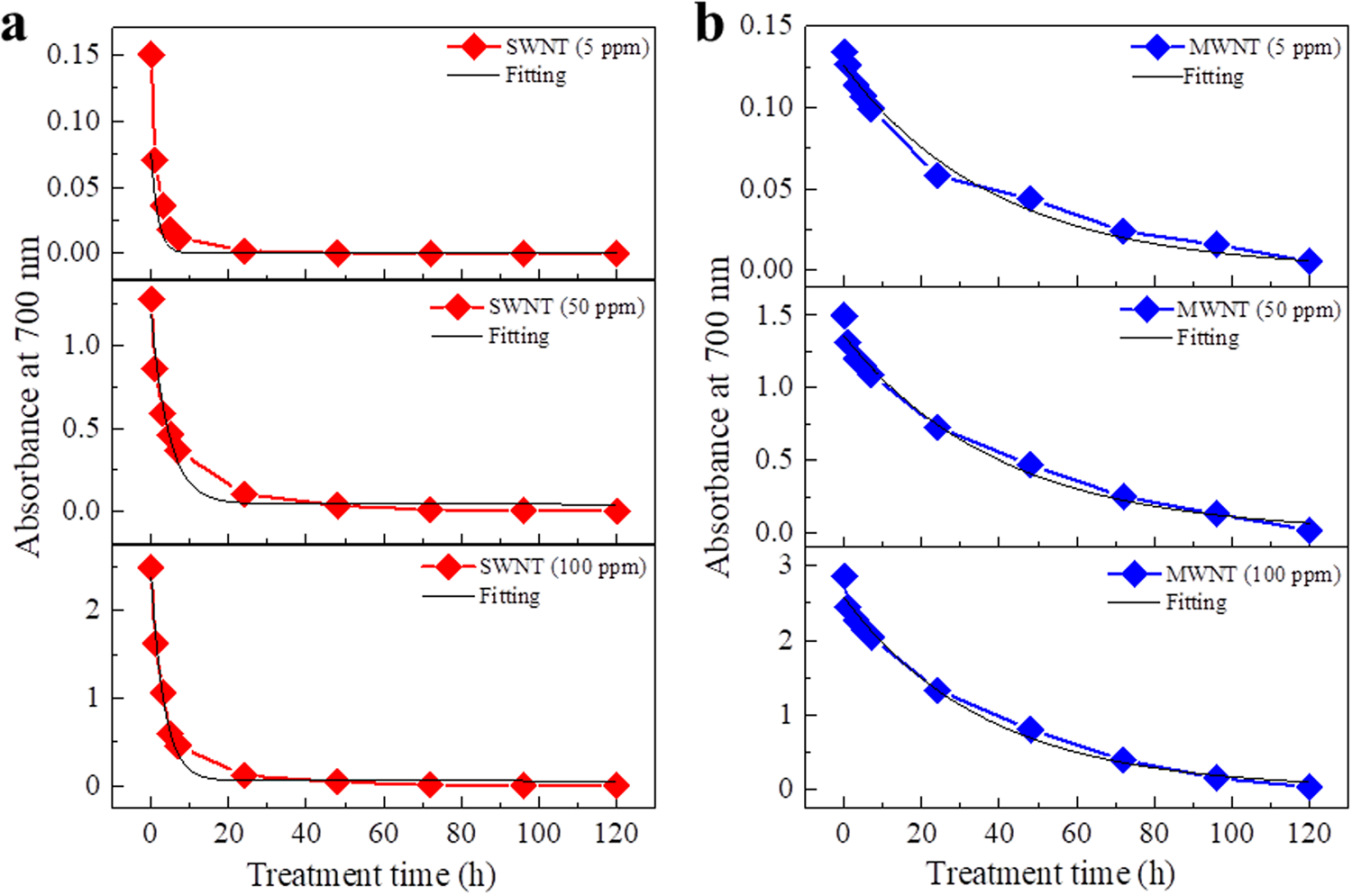
Estimation of the quantity of carbon nanotubes (CNTs) in aqueous dispersions following treatment with sodium hypochlorite solution. Red lines (**a**) and blue lines (**b**) indicate the absorbance at 700 nm for dispersions of SWNTs and MWNTs, respectively, after treatment for 0–96 h at 37 °C. Black lines represent curves fitted using the formula Y = Y_0_ + Ae^−x/t^ (Origin software).

The quantity of CNTs in dispersions was also estimated by measurement of the absorbance at 700 nm. The results showed that SWNTs were dramatically decreased in the first 1 h, then decreased to almost zero over 24 h (Fig. 2a). Degradation of MWNTs at all three concentrations was slower than that of SWNTs (Fig. 2b). In addition, the degradation rates for both SWNTs and MWNTs under the conditions used in this study fitted well to the first-order reaction rate curve Y = y_0_ + A_1_e^−kt^ (black curves in Fig. 2a,b), where Y is the concentration of CNTs, y_0_ is the final concentration of CNTs (approximately zero), A_1_ is the initial concentration of CNTs, k is reaction constant, and t is the reaction time. The half-value periods for complete degradation of SWNTs and MWNTs were estimated to be 1.25–2.5 h and 21–24 h, respectively.

**Figure 3:**
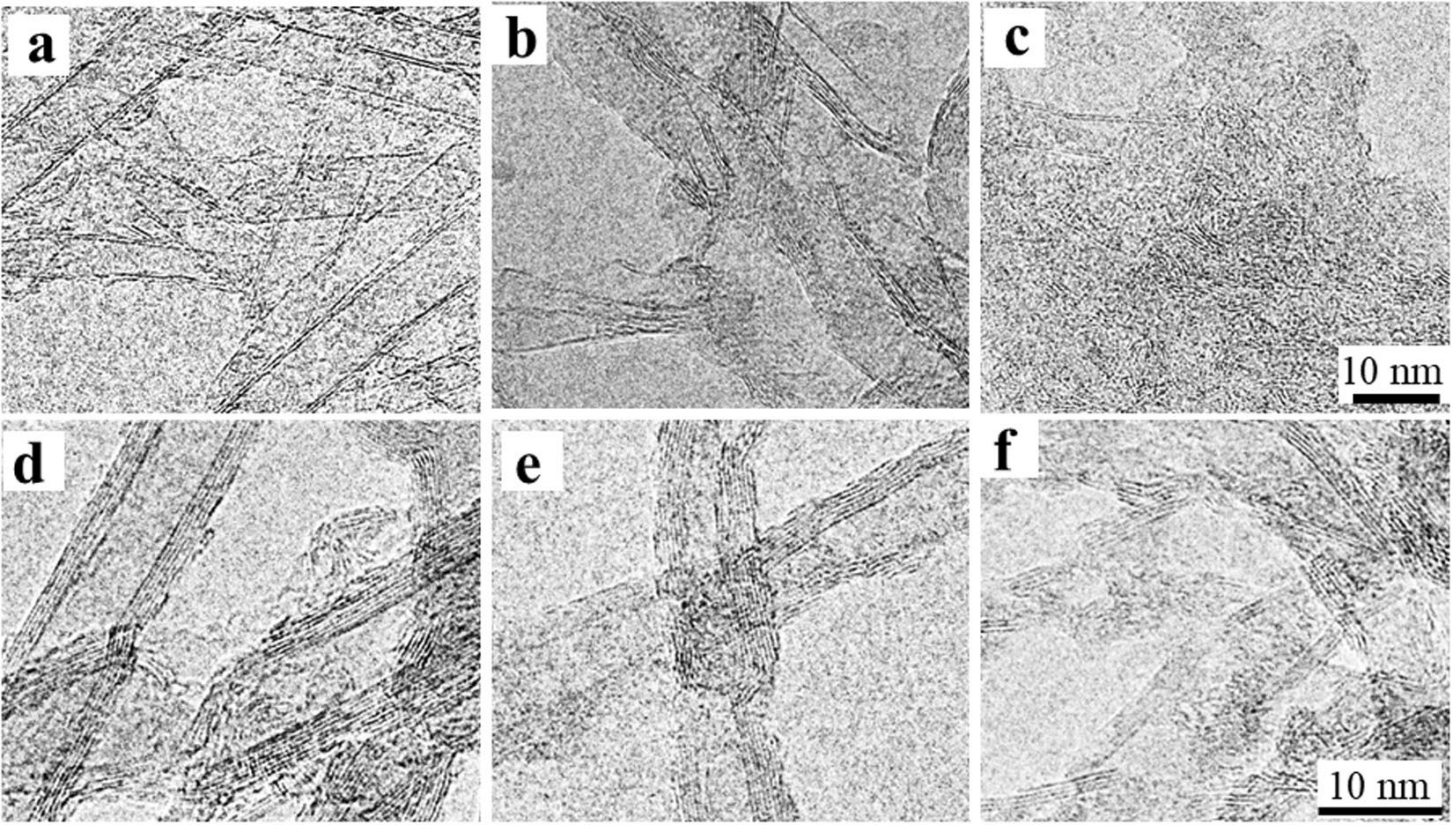
Transmission electron microscopy (TEM) images of SWNTs (**a**–**c**) and MWNTs (**d**–**f**) after treatment with sodium hypochlorite solution for 0, 3, and 24 h.

To investigate the structural changes of CNTs, CNT dispersion solutions (100 mg/L) were ultra-filtrated through a 10 kDa molecular weight cut-off membrane and rinsed with water before and after NaClO treatment for 1, 3, and 24 h. The obtained CNTs were analyzed by transmission electron microscopy (TEM) and Raman spectroscopy. TEM images showed that SWNT samples contained single walls, double walls (DWNTs) and a few MWNTs (Fig. 3a), in which DWNTs and MWNTs were impurities. After treatment with NaClO solution for 3 h, single-walled CNTs were no longer visible, and only DWNTs and thin graphite-like structures (or MWNTs) were observed (Fig. 3b). When the treatment time was extended to 24 h, the residues consisted of amorphous-like fragments, and only very few DWNTs or thin graphite-like fragments remained (Fig. 3c). For MWNT samples, structural changes after a 3 h treatment were not obvious (Fig. 3d,e), but a 24 h treatment resulted in residues with more amorphous-like structures, more greater damage to the walls of MWNTs (Fig. 3f).

**Figure 4:**
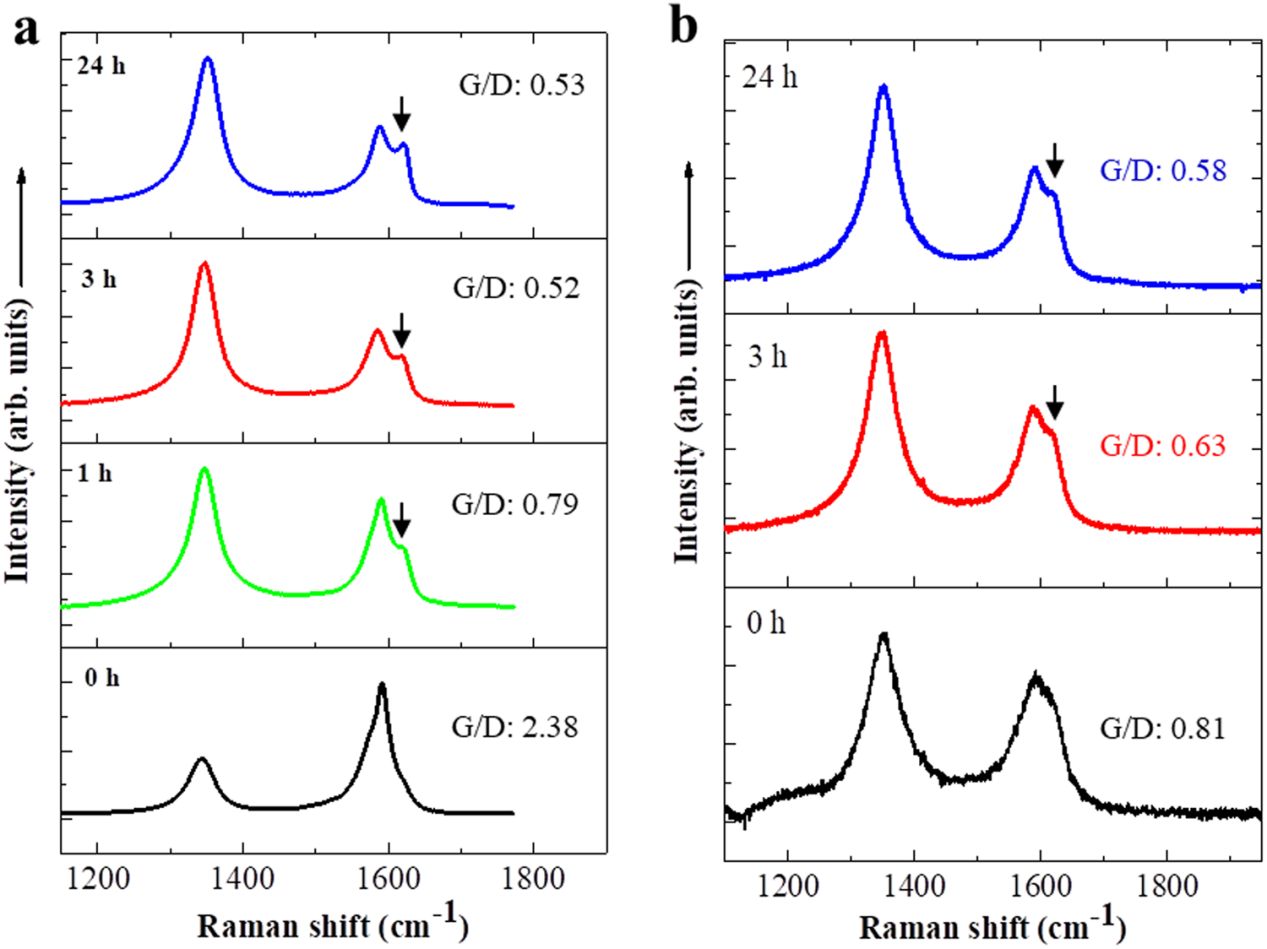
Raman spectra of SWNTs (**a**) and MWNTs (**b**) after treatment with sodium hypochlorite solution for 0, 1, 3, and 24 h.

These TEM observations indicate that SWNTs were degraded faster than MWNTs, consistent with the quantity change results described above (Figs 1 and 2). Raman spectra of SWNTs and MWNTs at 100 mg/L after NaClO treatment for 0–24 h (Fig. 4a,b) revealed G-band peaks (graphite) at 1591 cm^−1^, D-band (disordered) peaks at ~1350 cm^−1^, and D’ (second-order Raman scattering from D-band variation) peaks at ~1620 cm^−1^. The ratio of D-band to G-band intensities increased and D’ peaks at ~1620 cm^−1^ became more obvious in both samples after prolonged NaClO treatment, indicating increased wall damage and amorphous-like structure^31^ after NaClO treatment. In addition, when the treatment was longer than 120 h for both CNTs samples, no CNTs were detected by TEM or Raman measurements (data not shown).

**Figure 5:**
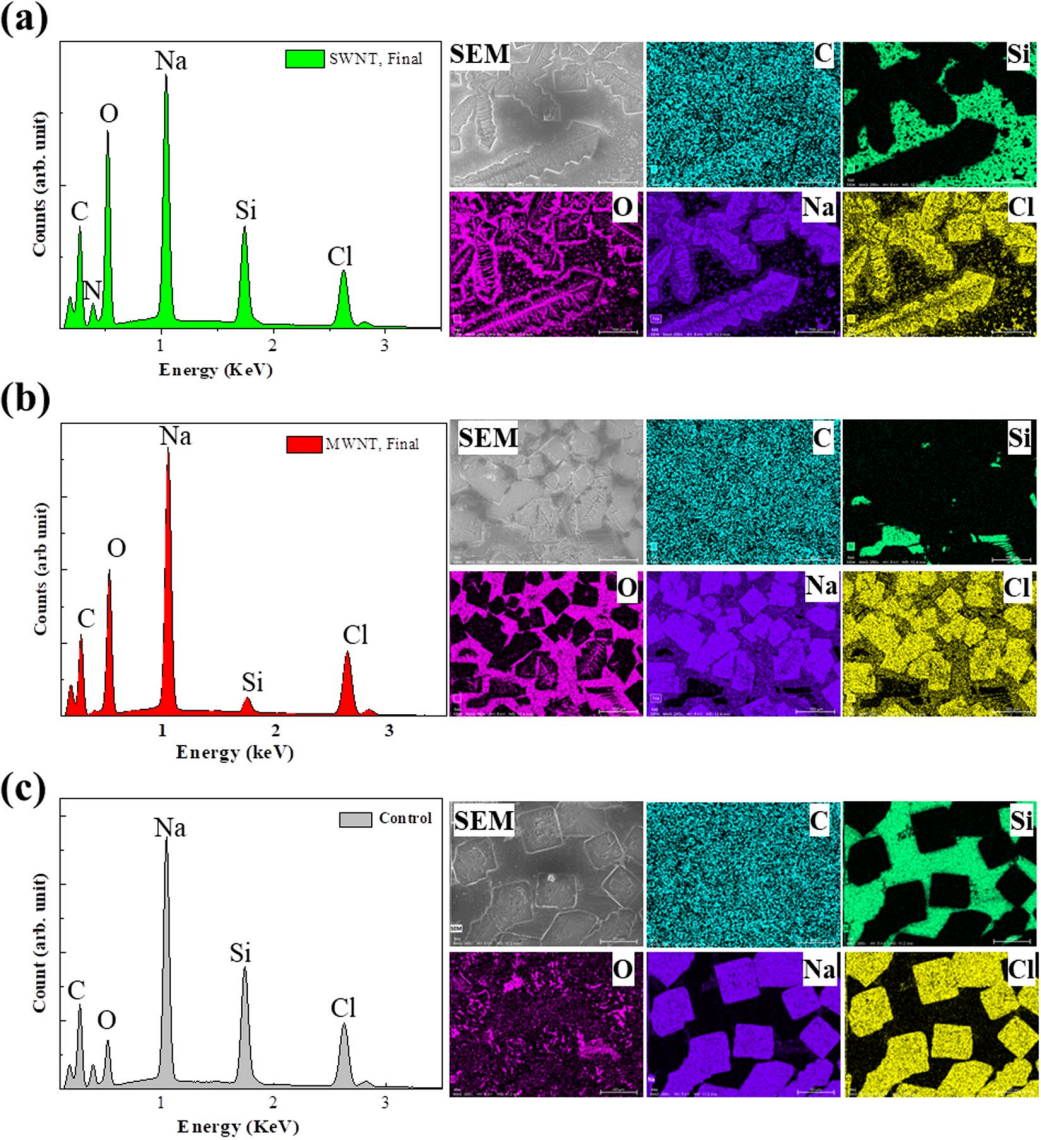
Scanning electron microscopy (SEM) images, energy dispersive X-ray spectroscopy (EDS) spectra and elemental mapping of C, Si, O, Na and Cl. The results are for SWNTs (**a**), MWNTs (**b**) and water controls (**c**) after sodium hypochlorite treatment for >120 h.

To confirm whether CNTs were completely degraded, we investigated the final colorless solution obtained from NaClO treatment after 120 h (hereafter referred to as the final solution). We heat-treated the obtained final solutions at 75 °C for 1 h to decompose most of NaClO, then analyzed them. First, we added CaCl_2_ solution to the resultant solution and found that the colorless transparent solution immediately changed into a white turbid solution, indicating the existence of carbonate ions in the final solution (Fig. SI-1). Second, we extracted aromatic hydrocarbons from the resultant solution using CH_2_Cl_2_, and materials dissolved in CH_2_CL_2_ were analyzed by mass spectrometry. No obvious peaks corresponding to aromatic hydrocarbon molecules were detected (Fig. SI-2), indicating the complete degradation of CNTs. One intermediate product with a mass-peak at 131 m/z (probably C_9_H_8_O) was observed in SWNTs treated for 24 h and in MWNTs treated for 96 h (Fig. SI-2a,c), but this peak almost disappeared after further treatment (Fig. S-2b). These results were slightly different from those obtained from enzyme-catalyzed degradation of SWNTs, in which several types of aromatic hydrocarbons were detected^25^. In addition, the final solutions after removal of ClO^−^ by adding HCl were placed on SiN wafers and dried for analysis by scanning electron microscopy (SEM) in combination with energy dispersive X-ray spectroscopy (SEM-EDS). The spectra indicated that there were elements of C, O, Cl, and Na in all samples, as well as Si and N of the SEM-plate of SiN wafer (Fig. 5). C-mapping images of the final solutions for SWNTs and MWNTs (Fig. 5a,b) were almost the same as that of the control sample (Fig. 5c), indicating that there was little or no C in the samples. The control specimen was prepared by mixing water with NaClO solution and treating with HCl. The results of semi-quantitative analysis confirmed that the percentage of C in all samples was almost the same. Although it is difficult to confirm the exact quantity of C in the samples due to background C from the SiN wafer and the EDS detector, we can confirm that the resultant products in final solutions were mainly NaCl from the 1:1 atom ratio of Na and Cl.

Taking into account the above results, we concluded that CNTs were completely removed by treatment with NaClO, and the resultant products of CNT degradation are [Na^+^], [Cl^−^], and [CO_3_^2−^] or [HCO_3_^−^]. NaClO is a powerful oxidizing agent in which [ClO^−^] donates an oxygen atom and its electrons to the species being oxidized, releasing a chloride ion (Cl^−^). Thus, the complete degradation of CNTs could be summarized by formula C_CNT_ + 2NaClO → CO_2_ + 2NaCl, where CO_2_ is partially or fully changed to [CO_3_^2−^] or [HCO_3_^−^] in aqueous solutions containing NaClO (pH 10).

To determine whether the method proposed in this study is suitable to all types of CNTs or other carbon nanomaterials (CNM), we treated six types of CNMs containing carbon nanohorns (CNHs), SWNTs, and MWNTs, which had not been acid treated but dispersed using bovine serum albumin assisted with sonication^31–33^. The results (Fig. S-3) showed that the six types of CNMs were completely degraded and that the degradation rates differed depending on CNM type. The degradation rates seemed to be dependent on the diameters of the CNTs in dispersion, in agreement with the results showing that the degradation rates of SWNTs were faster than those of MWNTs (Figs 1 and 2) and previous results showing that CNHs are degraded faster than MWNTs^29^. We suggest that the larger surface areas of CNTs with smaller diameters provide more reaction sites for reaction with NaClO, resulting in higher reaction rates for CNTs with smaller diameters than for those with larger diameters. We also found that CNTs dispersed by two different surfactants (data not shown) had almost the same degradation rates and were degraded completely. Together, the data indicate all CNT types in dispersion undergo complete degradation by hypochlorite, independent of whether they had been oxidized or not and the type of dispersant employed.

We also found that graphene oxide might be an intermediate product during SWNT degradation (Fig. 3), suggesting that CNT degradation might involve an unzipping process involving oxidization similar to the oxidation of CNTs by strong acid treatment^34–36^. However, the reaction rates with hypochlorite were much faster than those with strong acid treatment, and no obvious nanoribbon structures were found. In addition, because components in raw waste water are more complex than those in the CNT-dispersions employed in this study, the practicability of the proposed method needs to be confirmed using raw or simulated wastewater.

In summary, our results indicate that CNTs in aqueous solution such as wastewater can be removed completely using simple NaClO treatment. The degradation rates of CNTs with NaClO are dependent on their type: SWNTs were degraded faster than DWNTs, and MWNTs were degraded more slowly than SWNTs or DWNTs. CNTs were completely degraded and left no trace of hydrocarbon components, unlike degradation of CNTs by enzymatic oxidation. Although some issues remain to be clarified, such as the detailed mechanism of degradation of CNTs by NaClO, the relationship between the degradation rate and type of CNTs, and the influence of impurities as well as other substrates in the practical waste water on the degradation of CNTs, we believe that the results reported herein reveal a novel route for establishing a suitable method to eliminate CNTs from industrial wastewater, which is of significance in advancing the environmental sustainability of the CNT industry.

## Experimental Methods

### Preparation of CNT dispersions

For preparation of CNT aqueous dispersions, 50 mg of SWNTs produced by the super-growth method^37^ (SG-CNTs, Zeon Nanotechnology Co., Tokyo, Japan) and MWNTs (NC 7000, Nanocyl SA, Belgium) were dispersed and stirred in a 50 mL acidic solution of H_2_SO_4_/HNO_3_ (3:1) at 70 °C for 40 min. After treatment, CNT dispersions were immediately filtered through a 0.2 µm membrane, particles retained on the filter were resuspended in Milli-Q water, and this was repeated five times until the pH was neutral. The obtained CNTs were dispersed in 50 mL water and sonicated using a bath-type sonicator for 20 min to obtain CNT dispersions. The concentration of CNTs was estimated to be ~1 mg/mL.

### Degradation of CNTs by hypochlorite

The NaClO solution was prepared by dissolving 2.5 g NaClO∙5H_2_O powder (Wako, Japan) in water (10 mL), resulting in a NaClO concentration of ~11%. A 0.5 mL sample of NaClO solution was mixed with 5 mL of each of the CNT dispersions, resulting in a NaClO concentration of ~1.0%. The concentration of CNTs in dispersions was 5, 50 or 100 mg/L. After incubating CNT/NaClO mixtures at 37 °C for 0, 1, 3, 7, 24, 48, 72, 96, and 120 h, CNT dispersions were immediately analyzed using a UV-Vis-NIR spectrophotometer (Lambda 19, PerkinElmer Japan Co., Ltd.). The absorbance of each solution at 700 nm was recorded.

### Evaluation of structural changes in CNTs during degradation

SWNT and MWNTs were treated with NaClO for 0, 3, or 24 h, filtered using an ultra-centrifuge filter with a 10 kDa molecular weight cut-off, and washed with water five times. The obtained residues were then dispersed in water (0.2 mL) and a single drop of the resultant dispersion was placed on a glass slide and dried at room temperature overnight for Raman spectroscopy (LabRAM HR Evolution, Horiba, Japan). Measurements were taken at an excitation wavelength of 532 nm. The morphology of CNTs was observed using TEM (120 keV, 36 µA; 002B HRTEM, Topcon Corporation). Samples were prepared by placing a drop of each CNT dispersion on a copper grid and drying at room temperature.

### Analysis of the final products of CNTs after complete degradation

To assess the final products of CNTs after degradation with NaClO, the resultant final colorless transparent solutions (and the control solution prepared with water without CNTs) were heat-treated at 75 °C for 1 h to remove most of the NaClO, then analyzed. First, 0.5 mL CaCl_2_ solution (0.05 M) was added to 0.5 mL of the resultant solution to test for color changes. Second, the resultant solution (1 mL) was mixed with CH_2_Cl_2_ (1 mL) to extract any organic compounds, and extracts were subjected to liquid chromatography-mass spectrometry (JEOL, MStation, JMS 700, Tokyo, Japan).

For SEM combined with EDS using a Hitachi SU8220 and a BRUKER 5060FlatQUAD, respectively, HCl solution (100 µL, 6.5 M) was added to the final solution (1 mL) and heated at 70 °C for 2–3 min. The obtained solution was checked using a chlorine test-strip (WAP-ClO, Kyoritsu Chemical-Check Lab., Corp. Japan) to confirm that NaClO was completely removed. The pH of the solution was adjusted to neutral by adding NaOH solution, and the final solution was placed on a SiN wafer and dried at room temperature. SEM-EDS measurements were performed at an acceleration voltage of 5.0 kV and an emission current of 10 µA.

## Supplementary information

Supporting information
