# Supplementary material for: A Simple Method for Removal of Carbon Nanotubes from Wastewater Using Hypochlorite
Source: Sci Rep. 2019 Feb 4;9:1284. doi: 10.1038/s41598-018-38307-7 (PMC6362128; doi:10.1038/s41598-018-38307-7)

**Supporting information**

**A Simple Method for Removal of Carbon Nanomaterials from Wastewater Using Hypochlorite**

Minfang Zhang^1*^, Yinmei Deng^1^, Mei Yang^1^, Hideaki Nakajima^1^, Masako Yudasaka^1, 2^,

Sumio Iijima^2^, Toshiya Okazaki^1^

*^1^* *National Institute of Advanced Industrial Science and Technology (AIST),* *1-1-1 Higashi, Tsukuba, Ibaraki, 305-8565, Japan.*

*^2^* *Faculty of Science & Technology, Meijo University, 1-501 Shiogamaguchi, Tempaku-ku, Nagoya 468-8502, Japan.*


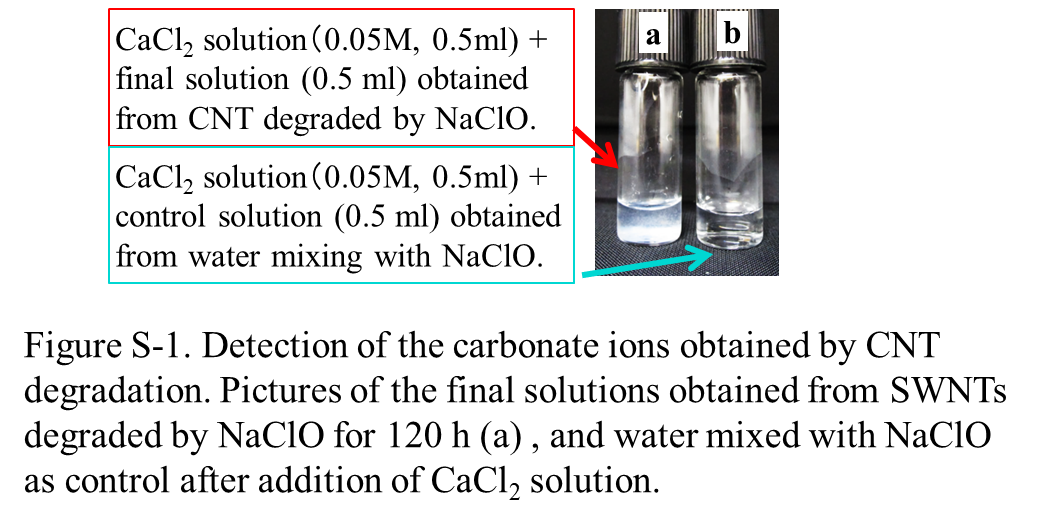


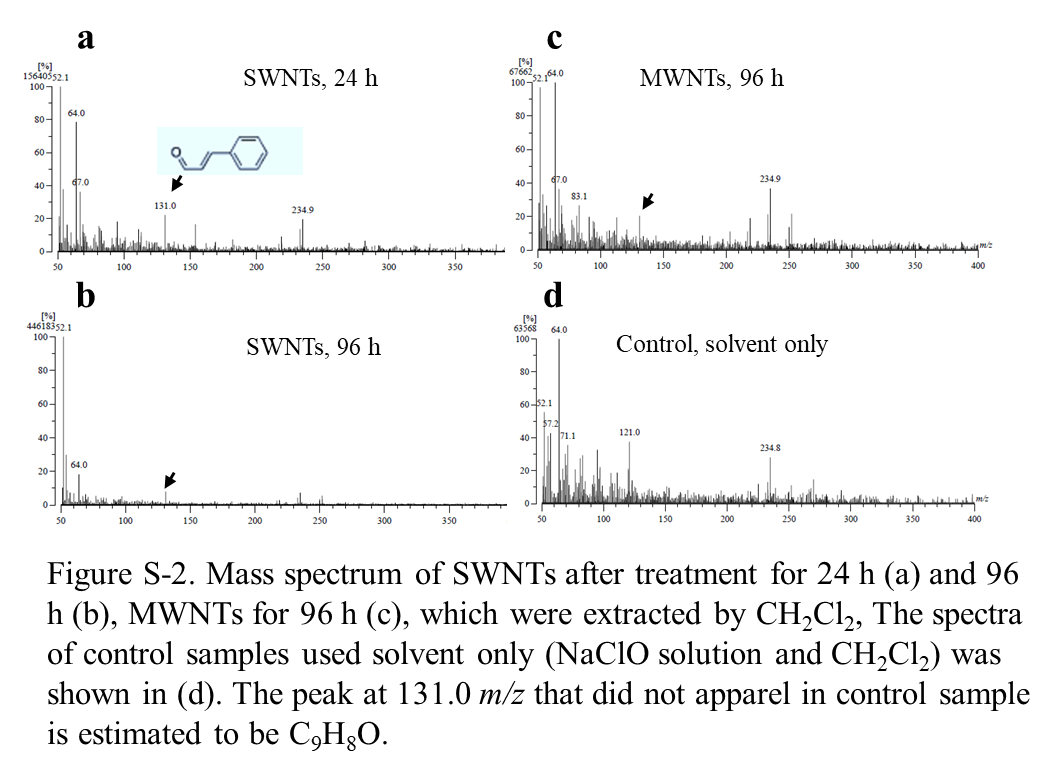

Supplement: Supplementary file 1 — Supporting information [file 41598_2018_38307_MOESM1_ESM.docx]
